# “We cobble together a storyline of system performance using a diversity of things”: a qualitative study of perspectives on public health performance measurement in Canada

**DOI:** 10.1186/s13690-022-00931-1

**Published:** 2022-07-29

**Authors:** Meghan O’Neill, Eric De Prophetis, Sara Allin, Andrew D. Pinto, Robert W. Smith, Erica Di Ruggiero, Robert Schwartz, Jasmine Pawa, Mehdi Ammi, Laura C. Rosella

**Affiliations:** 1 Population Health Analytics Lab, Dalla Lana School of Public Health, University of Toronto, Toronto, Ontario Canada; 2 Institute for Health Policy, Management and Evaluation, Dalla Lana School of Public Health, University of Toronto, Toronto, Ontario Canada; 3 North American Observatory on Health Systems and Policies, Dalla Lana School of Public Health, University of Toronto, Toronto, Ontario Canada; 4 Upstream Lab, MAP/Centre for Urban Health Solutions, Li Ka Shing Knowledge Institute, Unity Health Toronto, Toronto, Ontario Canada; 5 Division of Clinical Public Health, Dalla Lana School of Public Health, University of Toronto, Toronto, Ontario Canada; 6 Department of Family and Community Medicine, St. Michael’s Hospital, Toronto, Ontario Canada; 7 Department of Family and Community Medicine, Faculty of Medicine, University of Toronto, Toronto, Ontario Canada; 8 Social and Behavioural Health Sciences Division, Dalla Lana School of Public Health, University of Toronto, Toronto, Ontario Canada; 9 School of Public Policy and Administration, Carleton University, Ottawa, Ontario Canada; 10 Laboratory Medicine and Pathobiology, Temerty Faculty of Medicine, University of Toronto, Toronto, Canada; 11 ICES, Toronto, Ontario Canada; 12 Institute for Better Health, Trillium Health Partners, Mississauga, Ontario Canada

**Keywords:** Public health, Public health systems, Health system renewal; performance measurement system, Indicators, Qualitative analysis

## Abstract

**Background:**

There have been longstanding calls for public health systems transformations in many countries, including Canada. Core to these calls has been strengthening performance measurement. While advancements have been made in performance measurement for certain sectors of the health care system (primarily focused on acute and primary health care), effective use of indicators for measuring public health systems performance are lacking. This study describes the current state, anticipated challenges, and future directions in the development and implementation of a public health performance measurement system for Canada.

**Methods:**

We conducted a qualitative study using semi-structured interviews with public health leaders (*n* = 9) between July and August 2021. Public health leaders included researchers, government staff, and former medical officers of health who were purposively selected due to their expertise and experience with performance measurement with relevance to public health systems in Canada. Thematic analysis included both a deductive approach for themes consistent with the conceptual framework and an inductive approach to allow new themes to emerge from the data.

**Results:**

Conceptual, methodological, contextual, and infrastructure challenges were highlighted by participants in designing a performance measurement system for public health. Specifically, six major themes evolved that encompass 1) the mission and purpose of public health systems, including challenges inherent in measuring the functions and services of public health; 2) the macro context, including the impacts of chronic underinvestment and one-time funding injections on the ability to sustain a measurement system; 3) the organizational structure/governance of public health systems including multiple forms across Canada and underdevelopment of information technology systems; 4) accountability approaches to performance measurement and management; and 5) timing and unobservability in public health indicators. These challenges require dedicated investment, strong leadership, and political will from the federal and provincial/territorial governments.

**Conclusion:**

Unprecedented attention on public health due to the coronavirus disease 2019 pandemic has highlighted opportunities for system improvements, such as addressing the lack of a performance measurement system. This study provides actionable knowledge on conceptual, methodological, contextual, and infrastructure challenges needed to design and build a pan-Canadian performance measurement system for public health.

**Supplementary Information:**

The online version contains supplementary material available at 10.1186/s13690-022-00931-1.

## Background

Public health systems and services research is “a field of study that examines the organization, financing, and delivery of public health services and the impact of these services on public health” [1, 2]. Public health systems encompass both public-sector and non-governmental organizations [2]. Researchers aim to quantify the relationships between public health inputs and outcomes to add knowledge on the characteristics of strong performing public health systems [3]. One core aspect of this pursuit includes measuring and managing public health system performance [3]. Performance management uses performance measurement information to help set agreed-upon performance goals, allocate and prioritize resources, inform managers to either confirm or change current policy or program directions and report on the progress towards performance goals [4]. In recent decades considerable advancements have been made in performance measurement for certain sectors of the broader health system, including the health care sector (primarily focused on acute and primary health care) [5, 6]. However, effective use of indicators for measuring public health systems performance requires an approach that differs from the rest of the health care sector due to unique features of public health [7, 8].

The concept of a learning health system has been implemented to varying degrees across the United States and other jurisdictions internationally [9], primarily as a move towards value-based health care. While a learning health system has predominately been applied to health care contexts, there has been less attention and momentum in adopting this approach in public health systems which warrants greater attention. The movement towards a learning health system approach also represents an opportunity for increased collaboration between health care and public health, which may afford benefits such as improved workforce capacity, increased resources, and greater ability to rapidly leverage data to improve population health [10]. Broadly, a learning health system can be characterized as a system that harnesses data within and outside of the health system (i.e., public health, health care, and non-health sector organizations) to inform evidence-based decisions, with the goal of innovation and continuous quality improvement [11]. Public health systems are a subset of the broader health system that focuses on population health (as opposed to individuals) by means of disease prevention, health protection and health promotion [12].

Currently, resources necessary to collect, analyze, and rapidly use data collected within the public health system for performance measurement and management are limited and vary based on the scale (e.g., local, regional, national). At a national level, the primary platform for pan-Canadian health system performance comparisons is the Canadian Institute for Health Information’s health system indicators based largely on health care data. While population health surveillance and monitoring are among the core functions of public health, such functions are not designed for performance measurement and management purposes and therefore do not speak to how well the public health system is achieving its goals. Several recent reports outline intentions to make improvements to public health and health information technology systems that enable performance measurement. The 2020 and 2021 Chief Public Health Officer of Canada (CPHO)’s Reports highlighted ongoing plans to build pan-Canadian infrastructure and capacity for data collection [13, 14]. A priority action area defined in the 2021 CPHO report specifically included the development of performance measures that will hold governments accountable for achieving public health objectives and outcomes [14]. Lastly, the Public Health Agency of Canada’s Pan-Canadian Health Data Strategy (2021) was released, emphasizing the importance of reflecting on how we measure public health systems performance in Canada, which will allow us to understand how it is resourced and functioning [15]. A foundation for such efforts is a performance measurement and management system.

In a series of reports describing public health financing, governance, organization and workforce in Canada’s provinces and territories, we observed a paucity of indicators that measure the performance of the public health system specifically [16–18]. There are broader frameworks for performance indicators that encompass some components of public health (e.g., vaccination) and preventive care services (e.g., cancer screening); however, these indicators are usually embedded within a performance measurement framework for health care services [5, 14]. While the public health sector and health care sector (primarily focus on acute and primary care) are component parts of the broader health system, there are unique considerations and challenges to public health performance measurement and management. Existing approaches to performance measurement and management at local levels in Ontario, for example, have generated criticism. One such approach was the Ontario Public Health Standards, accompanied by accountability arrangements. These arrangements were found useful for compliance purposes instead of continuous quality improvement or rapid learning [8, 19, 20]. This study describes the current state, anticipated challenges, and future directions in developing and implementing a public health performance measurement system from the perspective of key public health systems leaders. The goal of this work is to provide actionable knowledge to establish a performance measurement system for public health systems in Canada.

## Methods

### Research methodology and modified framework

We adopted a qualitative phenomenological approach to this study [21] because our goal was to explore and understand the nature of participants’ own perspectives and experiences with performance measurement in public health systems as drawn from their lived experiences [21]. Our study questions were informed by a framework for performance measurement in public health systems developed by Turnock and Handler [22, 23].

### Participant selection

We recruited a purposive sample of nine key informants that consisted of individuals who were known opinion leaders in public health systems measurement [24]. We used a snowball sampling approach whereby study working group members developed an initial list of potential interviewees, who then identified other potential interviewees with deep knowledge and experience on the topic [21, 24]. Key informants were selected to ensure representation from professions and knowledge of different public health systems (i.e., we purposively selected two key informants who had expertise in Canada’s public health system and international systems). International experts were sought to add insights that may be applicable to the Canadian context.

#### Data collection

Key informants were interviewed via video conference by a single interviewer (MO) from July 15th to August 30th, 2021. A semi-structured interview guide was developed in consultation with the research team and the broader advisory group. A conceptual framework informed the structure of the interview guide for public health systems first developed by Turnock and Handler [22], which details a logic model according to the Donabedian dimensions, including structure, process, and outcomes. Handler and colleagues further adapted this model to include additional components, together with the mission/purpose of public health and the macro context in which public health systems operate [23]. Lastly, our key conceptual definitions and probes for gaps/challenges were informed by Derose and colleagues’ framework for local public health quality assessment [25] and other literature on public health systems measurement [3, 8]. Participants were invited through email and were sent a consent form and information letter prior to the interview. Verbal and/or written consent was obtained prior to initiating the interview. At the beginning of the interview, participants were asked to speak about their current and past positions related to public health systems measurement. We also asked participants to reflect on the current state of public health systems performance measurement, the collection of structure, process and outcome measures, and barriers and facilitators to the development of such a system (please see Table 1 for domains covered). Interviews were conducted in English and recorded and transcribed verbatim by a professional transcriptionist. Thematic analysis was conducted using *NVivo* (QSR International, Version 12).

**Table 1:** Interview guide informed by the conceptual framework for public heath system performance measurement

Domain	Interview Question
Participant introduction	Can you describe (based on current or past roles) what has been your involvement in public health systems performance measurement?
Current state of public health systems performance measurement in Canada	Currently, based on work in previous phase of his project, we’ve come to understand that there is not comprehensive public health performance measurement systems in Canada. Do you agree? If so, why do you think that is the case?
Structure, process, and outcome indicators	If you were tasked with improving the development, collection, measurement, and reporting of structure, process, and outcome indicators for public health systems, what would be the first step?
Conceptualization of structure, process, and outcome indicators	Among measures of structure, process and outcome that are related to the core public health functions^a^, what do you believe to be the most difficult to conceptualize or measure in assessing public health systems performance?
Barriers and facilitators to the development of a measurement system	For the last portion of our interview, I’d like to discuss the barriers and facilitators to the development of a public health performance measurement system. What do you believe to be the key barriers to developing and implementing a public health specific performance measurement systems?
Characteristics of a robust performance measurement system	What would a robust public health systems performance measurement look like to you?
Impact of a performance measurement system on the COVID-19 response	If we had public health systems performance management in place prior to COVID-19, do you believe it would have changed the public health response in Canada? In what ways could our response to COVID-19 have changed?

^a^Defined for key informants according to the 10 Essential Public Health Services by the Centers for Disease Control and Prevention [26]

### Data analysis

Data derived from the interviews were analyzed using descriptive and interpretive methods [27]. The qualitative analysis consisted of data reduction, display, and conclusion drawing. Thematic analysis [28], through the data reduction process, comprised of both deductive approaches to explore data for themes related to the indicators defined by the conceptual framework, complemented by an inductive approach to identify themes that emerged from the data. Directed content analysis [29] determined an initial coding guide [30] in advance of interviews based on the major domains identified in the conceptual framework. The qualitative analysis proceeded as follows: the interviewer (MO), with training and expertise in qualitative methods, independently coded three transcripts. The first set of transcripts was reviewed by another study team member (ED) and the study principal investigator (LR). The analysis team (MO, ED, LR) met to discuss emerging themes and concepts and to refine the coding guide following the first three interviews. Subsequently, the remainder of the interviews were coded by the interviewer and reviewed by the analysis team, with discrepancies or disagreements in coding resolved through discussion. As the analysis progressed, preliminary findings were presented to the broader advisory group in teleconference meetings to solicit feedback and refine the analysis. Criteria for evaluating qualitative research (i.e., credibility, transferability, dependability, and confirmability) were used to ensure qualitative rigour and to strengthen the comprehensiveness of our results [31]. This study is reported in line with the Consolidated Criteria for Reporting Qualitative Research checklist [32] (Supplemental file 1).

The results are organized according to a modified conceptual framework to measure the performance of the public health system by Handler and colleagues, which outlines the macro context of the public health system and how this relates to structure, process, and outcomes. Specifically, the framework encompasses 1) the mission/purpose of public health (i.e., goals of public health efforts and how they are operationalized), 2) the macro context of the public health system (i.e., the social, economic and political pressures that both, directly and indirectly, affect the public health system), 3) the structure of the public health system (i.e., the financial, organizational, informational, physical and human resources), and lastly, 4) the relationship between structure, process, and outcome indicators. This framework provides a unified conceptualization of the components of the public health system and how they interact, which is a useful model for guiding approaches to public health system performance.

## Results

Nine of the ten key informants responded and participated in our study (response rate = 90%). Two key informants worked in public health organizations internationally, while seven key informants worked in a Federal, Provincial or Territorial (FPT) public health system in Canada.

### The mission and purpose of public health

The challenge with clearly defining the key public health functions and services was a barrier highlighted by most key informants interviewed in this study. As a sector within the health system, key informants reflected that the functions and activities of public health have historically been defined differently and evolved to impact a wide range of health outcomes (e.g., infectious and non-infectious disease control). One key informant noted that although there have been advances in measuring the components of the public health systems in recent decades, consensus on the services, processes, and outcomes is a necessary step in realizing the potential of performance measurement for public health:

*“And I think there has been work…more work in recent years trying to develop those kinds of logic models for public health. But I think, first of all, really starting with getting that consensus of what public health does, and how it relates to those outcomes that we all want to achieve.”*

Key informants also relayed a tension between narrowly defining functions and services of the public health systems to facilitate measurement; however, they noted this limits the ability to capture the breadth of public health activities and impacts adequately. Key informants also noted that although organizations with a public health mandate carry out some public health services, other organizations also impact public health outcomes:

*“So ... you want to first really define what it is you’re trying to set up your performance measurement framework for. And if it is for achieving a set of population health goals, then that broader social determinants model makes sense. But then you’re not measuring just the impact of public health alone, right. And I think that’s where we get into the challenges. And in fact, ...the movement of a metric like life expectancy or general well-being of the population is going to be driven more by changes in education, social services, and a whole number of areas outside of the scope of public health.”*

As a result of ongoing challenges in clearly articulating the key functions and services, which tie back to the mission and purpose of public health, key informants noted that this impedes public health’s ability to secure steady and long-term funding (further described under *The Macro Context of Public Health Systems*):

*“I can say that from my own experience, which has been primarily federal, we have always had a lot of trouble #1 articulating what the system is. And people don’t really get it. But we’ve ...had a hard time advocating for public health investment in part because we’ve been unable to show outcome measures. And really, that is what will convince people that the public health system is where it needs to be or needs some investment. Those outcomes have been really, really difficult. And even now in the midst of the pandemic, it’s really hard for us to advocate. Whereas the health system can do that. They’ve got measures of beds,* etc. *that are very, very concrete. Whereas our outcome measures haven’t been standardized. We kind of cobble together a storyline of system performance using a diversity of things. So, I think for me, the challenge in system performance has been the lack of standardization, the lack of clarity in roles and responsibilities for who’s going to measure what, [and] how we’re going to report it.*

In addition to clarifying and clearly defining the functions and services, lack of role clarity and responsibility for reporting on different public heath indicators was also highlighted as a current barrier that needs to be addressed to realize the potential for performance measurement in public health.

### Macro context of public health systems

Key informants shared challenges related to tight budgets and one-time funding injections to public health that impede efforts to maintain performance measurement in the long-term and adapt a learning health system approach: *“I think it would need to have enough investment that this isn’t a one-time activity, but rather that we are taking the perspective of a learning system that has what it needs to learn…And it’s a constant cycle. So, I don’t see it as something that is static”.* One key informant emphasized the importance of advocating for a measurement system and how such a system could impact costs:

*“And then I think we would really need as a country to seek political will and support for the importance of [a performance measurement system], after which you can then seek funding that is akin to what is actually needed. Without that support, there’s just a lot of well-meaning people saying we need to do this. But you really need to sell it. There needs to be a storyline around it that has dollars attached to it because that is what helps. It needs to be a compelling storyline.”*

Key informants highlighted the reactionary nature of public health funding, whereby a one-time, large injection occurs after a serious public health threat has emerged. This is problematic for maintaining a robust and stable performance measurement system as the current funding approach does not facilitate the necessary public health infrastructure:

*“This came up in the Naylor report [a report on the National Advisory Committee on SARS and Public Health 2003]. It’s the funding cycle, right. So what happens, you have a big disaster, which was SARS. Now we’ve got another one, which is COVID. And then you have the reports. And everybody says, “Oh, this is terrible. You know, it’s a shortage of resources. So give them some more money.” Well, I was there in SARS, and the Naylor report, and that was the money. It was to build things like ICES and all of Panorama. Those were built with post-SARS money. But here we are 17 years later, and there’s no more money, right. So all the money comes after the outbreak, and then there’s no more money. So by the time the next outbreak comes, your systems are useless because you haven’t kept them up. So it’s not just funding, it’s consistency of funding that’s needed.”*

### The organizational structure of public health systems

Key informants reflected on how the organizational structure and delivery of public health programs and services within and across Canadian jurisdictions has encouraged a fragmented approach to reporting requirements and “*will depend on the configuration and who does what, and whether it’s within the public health service or whether it’s not.”*

One key informant described Canada’s public health systems as a *“cottage industry”* in which there is no standardized or pre-defined consistency in the organization and governance of public health across provinces and territories. When prompted about anticipated barriers to the successful implementation of a performance measurement framework, another key informant highlighted several challenges related to variable organization and governance and the impact this has on where accountability and reporting lies, and responsibilities for data collection and research:

*“It starts really with the organization and governance of public health… first starting with the constitution or split between the federal and provincial governments, with many of the public health services being under the health umbrella of constitutional responsibilities, especially with the provinces. And then some services and functions, particularly around statistics, which rests with the federal government as well as the international control research. So there’s quite a few of the things that would feed into public health performance measurement that are federal government responsibilities - statistics, research and so on. And that theme will probably come up over and over again. And then within the provinces and territories, there’s considerable variation in how public health is organized. And the variation has actually increased in recent years with regionalization of health services, amalgamation and so on... So that sort of variability means that from a governance perspective, it means that the sort of accountability for public health may rest with an independent agency like the Board of Health in Ontario, may be buried deep within an organization like Alberta Health Services”.*

Key informants stated that the desired state of performance measurement for the public health systems would require strong and ongoing commitments from FPT governments to strengthen capacity and infrastructure to rectify long standing issues with data linkage:

*“If we had a system in place with people engaged, and it was clear, for example, that we have major deficiencies on data linkage… across contact tracing, hospital data, death data, vaccine registry data. You know, if those limitations, we’ve been shining lights on them and so on, and people realized they were problems, and they could connect those deficiencies to some operational thing they were trying to do or decision they were trying to make, you know, I think it would have been a lot easier to get change. I mean we still don’t really have that problem solved. It’s been kind of pushed forward a little bit in some provinces, but not in a coherent way. And I still think they’re having limitations even federally”.*

Many key informants also noted that using existing and available data sources to answer questions about the performance of the public health systems is problematic and results in backwards-looking indicators. They emphasized the need to improve on the types of indicators collected instead of relying on the data we have available because you “*always end up with the widget stuff. You know, number of inspections, number of needles given, you know, that’s the stuff that…only tells a very, very tiny part of the picture”.* This sentiment was reinforced by many key informants cautioning against the adoption of performance indicators that are *“all skewed towards the stuff that’s easy to measure”* and the importance of re-envisioning how we measure performance in public health:

*“So I often go back to the famous quotation attributed to Einstein. What counts cannot necessarily be counted, and what can be counted does not necessarily count. In other words, what’s important can’t always be counted, and what you can count isn’t necessarily important. And I think that’s really a problem in public health”.*

Key informants highlighted that existing public health systems indicators are predominately focused on the components that are easy to measure, which risks under-emphasizing the importance of prevention (further described under *Timing and unobservability in public health indicators*).

### Accountability approaches to performance measurement and management

The most salient theme regarding the use of accountability via a performance measurement system was overwhelming resistance to attaching funding to performance. Key informants cautioned against financial incentives or penalties given that public health outcomes are impacted by factors and organizations outside the scope of organizations with a mandate for public health:

*“I would be very nervous about anything which had your finances tied to these metrics. Particularly because of this whole thing of the invisibility. What about the outbreak that never happened? You know what about the things you have no control over? So public health has critical, critical roles that you could absolutely shaft if their budgets are dependent on the wrong things.”*

While respondents agreed that there is considerable value in a performance measurement system for public health, there was a preference for assessing performance through rapid learning and innovation, achieved through a learning health system or balanced scorecard approach:

*“I guess the piece around innovation and learning, which is typically in a scorecard. I mean that’s part of the problem in public health, is that you have some places that feel like museums, frankly. Like you walk into them and going, “Oh, my, this is from some decades ago. And what happened that you didn’t evolve?… And so, you know, I think it’s an important aspect of a continuous improving system.”*

### Timing and unobservability in public health indicators

Most key informants noted that outcome indicators are commonly reported on, but there are several methodological challenges, including that the results of such indicators are difficult to disentangle from the effects of other contributing institutions and organizations outside the purview of public health. They highlighted issues measuring and quantifying the impacts of “non-events”:

*“So I think public health and the public health system and all of the partners within it, unlike the health system which can show within the course of a political cycle particular concrete measurement outcomes, we in the public health realm have been really challenged in order to do that. You know, stopping people from getting thrown into the lake rather than pulling them out later, that’s a much harder argument to prove at a political level. And then to seek funding to support the public health system, and, then by extension, the measurement of that system. So I think we’ve been very challenged in doing that.”*

Key informants noted that a strong performance measurement system should have a direct and strong link to pre-defined population outcomes. It is also important that such indicators be within the control of public health organizations. Finally, key informants cautioned about the long time lags necessary to observe meaningful changes in outcomes due to some public health efforts. This may be because the health outcome is insensitive to immediate policy or practice changes or other influences outside the scope of the public health.

## Discussion

This study reports on key informant perspectives on the current state, anticipated challenges, and future directions in the development and implementation of a public health performance measurement system for Canada. This study aimed to provide actionable insights to inform unique considerations and a comprehensive approach for the development of a public health-specific measurement system. Multiple conceptual, methodological, contextual and infrastructure challenges were highlighted by informants that can inform how and what is needed to build and implement such a system.

Our findings are well supported by a breadth of work both locally and internationally that has sought to describe the conceptual, methodological, and infrastructure challenges inherent in developing and implementing a performance measurement system for public health. For example, Schwartz and Deber [8] reviewed 55 public health measurement systems in high-income countries and assessed the extent to which they are being used for performance management. In public health, most measures serve informational purposes and are heavily focused on either process or outcome components of health care (e.g., 30-day surgical readmission rate). Furthermore, the data being collected in public health does not lend itself to rapid learning to the delivery of public health services [8], a concept referred to as the “performance measurement-management divide.”

Our qualitative study corroborates many of the findings from this review and adds context for why we see measurement and management efforts lacking public health sector indicators. Among them include difficulties discerning causal relationships between the actions of public health organizations and structure, process, and outcome measures, and the methodological challenges in accounting for time-lags between inputs and outcomes. Additionally, key informants emphasized the unique challenge faced in measuring the impact of prevention efforts. This was highlighted as a contributing factor to difficulties in advocating for funding and an over-reliance on indicators that are easy to measure. Many of the identified barriers for optimal health information systems, including tight budgets and one-time funding injections, limited human resource capacity due to cuts to public health, or political will for a performance measurement system, create substantial challenges for developing and sustaining performance measurement systems that may make the benefits of prevention more visible. These barriers are not unique to Canada’s health system and have been observed in other health systems including in the United States [33] and Europe [34].

The findings from this paper highlight the need for FPT governments to commit to supporting performance measurement through sustained investment in infrastructure that would support a learning health system [11]. With the origins of a learning health system largely developed in the context of the United States predominately privately funded health system, Menear et al. [35], developed a conceptual framework for how a learning health system may be applied in the Canadian context. Amongst a series of pillars include critical infrastructure, systems and resources.

Behn and colleagues [36] emphasize that performance measurement is not an end but a means to an end (i.e., performance management) and that the purpose is context-specific. The structure and goal of the performance management system should be explicitly communicated. In addition to commitments to supporting performance measurement through investment in infrastructure and personnel, FPT governments should clearly articulate what is meant by accountability, which encompasses answering “for what, by whom and to whom and how” [37]. Boland and Fowler [38] provide a useful matrix that is broken down into the source of the control (internal or external) and the resulting action, including positive (supportive and formative) or negative (punishment and summative). The use of performance indicators in public health has been used both for accountability and for determining future resource allocation. Freeman and colleagues [39] highlight how this approach can be problematic, whereby organizations deemed to be performing well are afforded additional resources and organizations performing poorly must demonstrate improvement with even fewer resources. In agreement with this view, key informants in our study cautioned against tying funding to a performance measurement system and instead preferred a learning health system approach [11]. Lastly, this work supports the position that performance measurement should be funded and sustained long term, which will facilitate rapid learning and continuous quality improvement.

### Strengths and limitations

Our paper provides a timely and comprehensive examination of multiple issues meriting attention in order to build an effective public health performance measurement system in Canada. Our data analysis is strengthened by the robustness of our qualitative approach, applying the principles of credibility, dependability, confirmability and transferability [31]. To ensure our interpretations were reflective of the experiences of key informants, we relayed our qualitative summary back to key informants to validate or provide feedback. Another strength of the present study includes the approach used to select key informants, which resulted in a sample of thought leaders with different professional experiences and roles in public health systems both locally and internationally. To our knowledge, this is the first qualitative study with perspectives of key public health leaders with a direct focus on performance measurement in Canada, which underscores the novelty and insights afforded by this work. One limitation of this study includes the relatively modest sample of key informants that may not comprehensively cover the breadth of considerations for the implementation of a performance measurement system. Specifically, we did not interview key informants from Indigenous-led public health organizations, which represents a gap in the literature. Future research should also solicit perspectives from public health leaders in other Provinces and Territories.

## Conclusion

There have been several calls to transform public health systems in Canada, catalyzed by the major gaps brought to light by the COVID-19 pandemic. Core to the needed reforms is data and information systems needed to carry out the public health functions and measure performance to identify gaps and make improvements. This study provides actionable knowledge on conceptual, methodological, and infrastructure insights required for the design and building of a public health pan-Canadian performance measurement system. We identified several gaps in public health indicators and the scope of proposed public health measures required for performance measurement that stem from longstanding challenges in the structure and funding of the public health system. Dedicated investment, committed leadership and governance, and political will from the FPT governments to build sustainable performance measurement infrastructure are required. Once the system is built, there are additional considerations and engagement needed to inform how such a system is operationalized and used to inform rapid learning and change.

## Supplementary Information

**Additional file 1:** COREQ Checklist.

## Data Availability

Data sharing is not applicable to this article as no datasets were generated or analysed during the current study.

## Abbreviations

FPT: Federal, Provincial, and Territorial
COVID-19: Coronavirus disease 2019
CPHO: Chief Public Health Officer of Canada
REB: Research ethics board
SARS: Severe acute respiratory syndrome
