# Supplementary material for: “We cobble together a storyline of system performance using a diversity of things”: a qualitative study of perspectives on public health performance measurement in Canada
Source: Arch Public Health. 2022 Jul 29;80:177. doi: 10.1186/s13690-022-00931-1 (PMC9335461; doi:10.1186/s13690-022-00931-1)
Supplement: Supplementary file 1 — Additional file 1: COREQ Checklist. [file 13690_2022_931_MOESM1_ESM.docx]

**COREQ Checklist**

| **Item No.** | **Item** | **Guide Questions/Descriptions** | **Response** |
| --- | --- | --- | --- |
| **Domain 1: Research team and reflexivity** | | | |
| **Personal Characteristics** | | | |
| 1. | Interviewer/facilitator | Which authors conducted the interview or focus group? | MO. In one instance a second member of the research team was present (SA). |
| 2. | Credentials | What were the researcher's credentials? *E.g. PhD, MD* | MO: Masters of Public Health. |
| 3. | Occupation | What was their occupation at the time of the study? | MO is a Research Officer and Epidemiologist. |
| 4. | Gender | Was the researcher male or female? | MO is female. |
| 5. | Experience and training | What experience or training did the researcher have? | MO has previous qualitative research training gained through a combination of education and over four years of experience using qualitative methods. |
| **Relationship with participants** | | | |
| 6. | Relationship established | Was a relationship established prior to study commencement? | Participants were recruited through pre-existing relationships with the study team and broader working group. There was no previous relationship between the interviewer and the participant. |
| 7. | Participant knowledge of the interviewer | What did the participants know about the researcher? e*.g. personal goals, reasons for doing the research* | The participants were made aware of the background and aim of the study by the researchers prior to contacting them to arrange an interview time. |
| 8. | Interviewer characteristics | What characteristics were reported about the interviewer/facilitator? e.g. *Bias, assumptions, reasons and interests in the research topic* | For the purposes of reflexivity, after each interview, the interviewer debriefed to document ways the interview and their understandings drawn from it may have been influenced by their beliefs/assumptions/biases. |
| **Domain 2: Study design** | | | |
| **Theoretical framework** | | | |
| 9. | Methodological orientation and Theory | What methodological orientation was stated to underpin the study? *e.g. grounded theory, discourse analysis, ethnography, phenomenology, content analysis* | Thematic analysis and phenomenology. |
| **Participant selection** | | | |
| 10. | Sampling | How were participants selected? *e.g. purposive, convenience, consecutive, snowball* | Recruitment included a combination of purposive (maximum variation) sampling and snowball sampling. |
| 11. | Method of approach | How were participants approached? e*.g. face-to-face, telephone, mail, email* | Participants were recruited by email. |
| 12. | Sample size | How many participants were in the study? | 9 |
| 13. | Non-participation | How many people refused to participate or dropped out? Reasons? | We invited 10 particpants to particpate in the study. Out of the 10 participants, 9 completed the interview resulting in a response rate of 90%. |
| **Setting** | | | |
| 14. | Setting of data collection | Where was the data collected? e*.g. home, clinic, workplace* | Data was collected at home by videoconferencing software. |
| 15. | Presence of non-participants | Was anyone else present besides the participants and researchers? | Not to our knowledge. |
| 16. | Description of sample | What are the important characteristics of the sample? *e.g. demographic data, date* | Participant geography and role. |
| **Data collection** | | | |
| 17. | Interview guide | Were questions, prompts, guides provided by the authors? Was it pilot tested? | Interviews were conducted using a semi-strutured interview guide created by the authors which was informed by the literature. The interview guide was piloted with members of the research team and recieved iterative feedback from the working group. |
| 18. | Repeat interviews | Were repeat interviews carried out? If yes, how many? | No. |
| 19. | Audio/visual recording | Did the research use audio or visual recording to collect the data? | All interviews were audio-recorded and transcribed verbatim. |
| 20. | Field notes | Were field notes made during and/or after the interview or focus group? | Feild notes were made during and after the interviews to record any notable comments or interviewer reflections across participants. |
| 21. | Duration | What was the duration of the interviews or focus group? | Interviews lasted approximately 60 minutes. |
| 22. | Data saturation | Was data saturation discussed? | Yes, we considered data saturation at the recruitment phase with the assumption that approxiately 8-10 participants would result in reoccuring and reinforcing reflections on themes. |
| 23. | Transcripts returned | Were transcripts returned to participants for comment and/or correction? | Transcripts that were not yet thematically analyzed were not returned to participants for comment and/or correction. |
| **Domain 3: Analysis and findings** | | | |
| **Data analysis** | | | |
| 24. | Number of data coders | How many data coders coded the data? | Two (MO, ED) |
| 25. | Description of the coding tree | Did authors provide a description of the coding tree? | No. |
| 26. | Derivation of themes | Were themes identified in advance or derived from the data? | Themes were both idenfitied in advance and were allowed to evolve from the data. |
| 27. | Software | What software, if applicable, was used to manage the data? | *NVivo* (QSR International, Version 12). |
| 28. | Participant checking | Did participants provide feedback on the findings? | Yes, member checking was conducted for all interviews whereby the thematic summary was shared with participants to verify our interpretations resonated with their experiences. Two particpants reviewed the summary and validated our analysis. |
| **Reporting** | | | |
| 29. | Quotations presented | Were participant quotations presented to illustrate the themes/findings? Was each quotation identified? e*.g. participant number* | Yes. |
| 30. | Data and findings consistent | Was there consistency between the data presented and the findings? | Yes. |
| 31. | Clarity of major themes | Were major themes clearly presented in the findings? | Yes. |
| 32. | Clarity of minor themes | Is there a description of diverse cases or discussion of minor themes? | Yes. |
